# Supplementary material for: From sequence to enzyme mechanism using multi-label machine learning
Source: BMC Bioinformatics. 2014 May 19;15:150. doi: 10.1186/1471-2105-15-150 (PMC4229970; doi:10.1186/1471-2105-15-150)
Supplement: Additional file 2 — Java code of ml2db. Additional file ml2db_code.tar.gz contains the Java source code to run the multi-label machine learning experiments and save the results to database. The code’s Javadoc is included. [file 1471-2105-15-150-S2.zip › additional file 2/ml2db/utils/doc/uk/ac/standrews/utils/main/setutils/SupersetsManager.html]

SupersetsManager


JavaScript is disabled on your browser.


- Overview
- Package
- Class
- Use
- Tree
- Deprecated
- Index
- Help

- Prev Class
- Next Class

- Frames
- No Frames

- All Classes

- Summary:
- Nested |
- Field |
- Constr |
- Method

- Detail:
- Field |
- Constr |
- Method


uk.ac.standrews.utils.main.setutils

## Class SupersetsManager

- java.lang.Object
- - uk.ac.standrews.utils.main.setutils.SupersetsManager

- ---

    

  ```
  public class SupersetsManager
  extends java.lang.Object
  ```

  A class to identify the unique supersets among sets of interpro signatures. A
  set is a subset of a given superset if all its elements belong to the
  superset. For example: a-b, or a-c, or b-d are subsets of a-b-c-d, but a-e is
  not. A set is always a subset of itself.
  Beware not a very generic class (null are represented as '-1'), if -1 has a
  meaning in your data, modify this class accordingly.

  Version:
  :   16 Mar 2011

  Author:
  :   Luna De Ferrari luna.deferrari-at-ed.ac.uk

- - ### Field Summary

    Fields

    | Modifier and Type | Field and Description |
    | --- | --- |
    | `static boolean` | `m_debug` |
  - ### Constructor Summary

    Constructors

    Constructor and Description

    `SupersetsManager(java.lang.String sqlQuery,
    DbManager dbManager,
    java.lang.String separator)`

    `SupersetsManager(java.util.TreeMap<java.lang.String,java.lang.String> setsIdsElements,
    java.lang.String separator)`
  - ### Method Summary

    Methods

    | Modifier and Type | Method and Description |
    | --- | --- |
    | `java.util.TreeSet<Set>` | `createSupersets()` |
    | `void` | `generateSupersets()` |
    | `java.lang.String` | `getSeparator()` |
    | `java.util.TreeSet<Set>` | `getSets()` |
    | `java.util.TreeSet<Set>` | `getSupersets()` |
    | `java.lang.String` | `getSupersetsCSV()` Get a comma separated list of [superset, subset] couples. |
    | `OneToManyMap` | `getSupersetsIds()` |
    | `java.lang.String` | `getSupersetsIdsCSV()` Get a comma separated list of [superset id, subset id] couples. |
    | `static void` | `main(java.lang.String[] args)` |

    - ### Methods inherited from class java.lang.Object

      `equals, getClass, hashCode, notify, notifyAll, toString, wait, wait, wait`

- - ### Field Detail


    - #### m\_debug

      ```
      public static final boolean m_debug
      ```

      See Also:
      :   Constant Field Values
  - ### Constructor Detail


    - #### SupersetsManager

      ```
      public SupersetsManager(java.lang.String sqlQuery,
                      DbManager dbManager,
                      java.lang.String separator)
      ```

      Parameters:
      :   `sqlQuery` - to get the id + string of set elements divided by the
          separator
      :   `dbConnectionPath` -
      :   `separator` -


    - #### SupersetsManager

      ```
      public SupersetsManager(java.util.TreeMap<java.lang.String,java.lang.String> setsIdsElements,
                      java.lang.String separator)
      ```

      Parameters:
      :   `sqlQuery` - to get the id + string of set elements divided by the
          separator
      :   `dbConnectionPath` -
      :   `separator` -
  - ### Method Detail


    - #### main

      ```
      public static void main(java.lang.String[] args)
      ```


    - #### createSupersets

      ```
      public java.util.TreeSet<Set> createSupersets()
      ```


    - #### generateSupersets

      ```
      public void generateSupersets()
      ```


    - #### getSeparator

      ```
      public java.lang.String getSeparator()
      ```


    - #### getSets

      ```
      public java.util.TreeSet<Set> getSets()
      ```


    - #### getSupersets

      ```
      public java.util.TreeSet<Set> getSupersets()
      ```


    - #### getSupersetsCSV

      ```
      public java.lang.String getSupersetsCSV()
      ```

      Get a comma separated list of [superset, subset] couples.

      Returns:
      :   a comma separated list of [superset, subset] couples.


    - #### getSupersetsIds

      ```
      public OneToManyMap getSupersetsIds()
      ```


    - #### getSupersetsIdsCSV

      ```
      public java.lang.String getSupersetsIdsCSV()
      ```

      Get a comma separated list of [superset id, subset id] couples.

      Returns:
      :   a comma separated list of [superset id, subset id] couples.


- Overview
- Package
- Class
- Use
- Tree
- Deprecated
- Index
- Help

- Prev Class
- Next Class

- Frames
- No Frames

- All Classes

- Summary:
- Nested |
- Field |
- Constr |
- Method

- Detail:
- Field |
- Constr |
- Method
